# Single Leg Cycling Offsets Reduced Muscle Oxygenation in Hypoxic Environments

**DOI:** 10.3390/ijerph19159139

**Published:** 2022-07-26

**Authors:** Shane Draper, Tyler Singer, Cody Dulaney, John McDaniel

**Affiliations:** 1 Department of Exercise Science and Outdoor Recreation, Utah Valley University, Orem, UT 84058, USA; shaned@uvu.edu; 2 Department of Exercise Science, Fairmont State University, Fairmont, WV 26554, USA; tyler.singer@fairmontstate.edu; 3 Department of Fitness and Wellness Leadership, State University of New York Plattsburgh, Plattsburgh, NY 12901, USA; cdula002@plattsburgh.edu; 4 Department of Exercise Science, Kent State University, Kent, OH 44242, USA; 5 Advanced Platform Technology Center, VA Northeast Ohio Healthcare System, Cleveland, OH 44106, USA

**Keywords:** blood flow, hypoxia, small muscle mass exercise, tissue oxygen saturation, muscle oxygenation, cycling, tissue perfusion, cardiovascular

## Abstract

The intensity of large muscle mass exercise declines at altitude due to reduced oxygen delivery to active muscles. The purpose of this investigation was to determine if the greater limb blood flow during single-leg cycling prevents the reduction in tissue oxygenation observed during traditional double-leg cycling in hypoxic conditions. Ten healthy individuals performed bouts of double and single-leg cycling (4, four-minute stages at 50–80% of their peak oxygen consumption) in hypoxic (15% inspired O^2^) and normoxic conditions. Heart rate, mean arterial pressure, femoral blood flow, lactate, oxygenated hemoglobin, total hemoglobin, and tissue saturation index in the vastus lateralis were recorded during cycling tests. Femoral blood flow (2846 ± 912 mL/min) and oxygenated hemoglobin (−2.98 ± 3.56 au) during single-leg cycling in hypoxia were greater than double-leg cycling in hypoxia (2429 ± 835 mL/min and −6.78 ± 3.22 au respectively, *p* ≤ 0.01). In addition, tissue saturation index was also reduced in the double-leg hypoxic condition (60.2 ± 3.1%) compared to double-leg normoxic (66.0 ± 2.4%, *p* = 0.008) and single-leg hypoxic (63.3 ± 3.2, *p* < 0.001) conditions. These data indicate that while at altitude, use of reduced muscle mass exercise can help offset the reduction in tissue oxygenation observed during larger muscle mass activities allowing athletes to exercise at greater limb/muscle specific intensities.

## 1. Introduction

Historically, endurance athletes have attempted to improve sea level performance through a variety of different means. The idea that training at altitude can augment sea level endurance performance gained widespread acceptance among athletes [1,2,3,4,5,6,7,8]. However, there has been debate over whether there are additional benefits to altitude training [9,10,11,12,13] due to exercise intensity limitations imposed by reduced blood oxygen saturation at altitude as well as debate on the mechanisms responsible for such potential enhanced endurance performance [14]. As a result, the live high–train low paradigm [15,16,17] is a more optimal method to induce the favorable cardiovascular, metabolic, and respiratory adaptations of altitude exposure while circumventing decreased training intensity associated with exercise at altitude. However, for many athletes who live at altitude, the live high–train low paradigm is not feasible due to time constraints, financial resources, and easily accessible training sites at lower altitudes. Thus, to optimize performance for these individuals it is imperative to find an alternative way to train at sea level exercise intensities while living at altitude.

Single-leg cycling is a reduced muscle mass exercise that has been used as both a rehabilitative as well as training modality. Single-leg cycling has been reported to generate greater leg-specific work rates [18,19,20,21,22,23] due to greater blood flow and oxygen delivery to active muscles [24]. This increased capacity to perform limb-specific work or power during single-leg cycling and other reduced muscle mass activities (i.e., knee extension) leads to greater muscle specific adaptations in highly trained and diseased populations [19,25,26,27] compared to double-leg cycling and other larger muscle mass training modalities. Thus, elevated limb specific blood flow associated with single-leg cycling may also benefit exercise training at altitude. It has previously been reported that blood saturation, VO_2_ peak, and peak power are reduced in single-leg cycling in hypoxia [28], however, it is still unknown if single-leg cycling can be used to at least partially offset the likely greater reduction in blood saturation and subsequent performance normally seen with traditional double-leg cycling. If so, this will allow athletes to exercise at the greater muscle-specific work rates typically associated with sea level training.

Therefore, the purpose of this study was twofold. In the first study, we wanted to compare the physiological responses to double-leg cycling in normoxic and hypoxic conditions. We then wanted to assess whether transitioning to single-leg cycling in the hypoxic condition would minimize any observed reductions in tissue oxygenation. We hypothesized that tissue oxygen saturation would be reduced during submaximal double-leg cycling in hypoxia compared to normoxia. We also hypothesized that single-leg cycling will result in a greater hyperemic response in the active limb, thereby minimizing the reduction in tissue oxygenation in hypoxia. In the second study, we hypothesized that similar to submaximal exercise, a short bout of high-intensity exercise in hypoxia would compromise tissue oxygenation and reducing the muscle mass via single-leg cycling would restore tissue oxygenation similar to that observed during normoxia.

## 2. Materials and Methods

### 2.1. Participants

A statistical power analysis was performed for sample size estimation using G*Power statistical power software (G*Power, Heinrich-Heine-University, Dusseldorf, Germany). The effect size for blood flow and hemoglobin was considered to be large (0.40) based on Cohen’s f criteria with an alpha of (0.05) and a power of (0.80) the projected sample size needed with this effect size was 10. Ten healthy and recreationally active individuals were recruited to participate in phase 1 (5 males and 5 females; height: 177.5 ± 8.0 cm; weight: 74.2 ± 13.2 kg; VO_2_ peak: 43.0 ± 8.3 mL·min·kg; 25 ± 3 years) as well as phase two (6 males and 4 females; height: 175.7± 10 cm; weight: 74.9 ± 15.4 kg; VO_2_ peak: 44.4 ± 7.5 mL·min·kg, age: 25 ± 3 years) of this investigation. Participants selected for either phase were not obese, currently taking any medications, nicotine users, diagnosed with cardiovascular, respiratory, or metabolic diseases and were not exposed to an altitude above 2500 m within two months prior to participation in the study. Participants self-reported to be moderately active based on the American College of Sports Medicine (ACSM) exercise participation health screening guidelines [29]. For both phases of this study, participants were required to visit the laboratory on three separate occasions. This study was approved by the Kent State University Institutional Review Board (IRB log number 17–289).

### 2.2. Study Design

**Figure 1:**
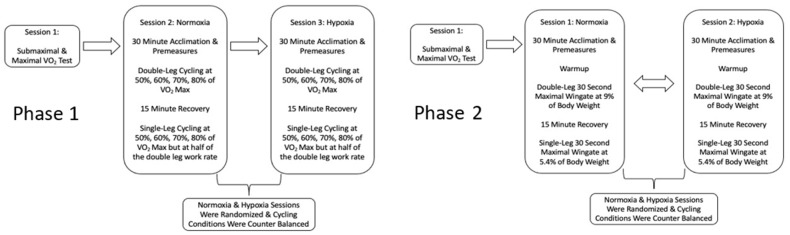
Timeline for phase 1 and 2.

Subjects were recruited to participate in either or both phases of this investigation. Each phase consisted of a preliminary testing session followed by two data collection sessions that were separated by 2–7 days. Phase one (Figure 1) consisted of three submaximal cycling conditions (double-leg normoxia, double-leg hypoxia and single-leg hypoxia) each with 4 levels of cycling intensity (3 × 4 repeated measures). Phase two (Figure 1) consisted of 3 maximal cycling conditions (double-leg normoxia, double-leg hypoxia and single-leg hypoxia). For both phases of this investigation the normoxic and hypoxic conditions were assigned in a counterbalanced order to visits 2 and 3.

### 2.3. Visit 1: Prescreening and Baseline Testing

During the first session participants completed an informed consent and a health history questionnaire. Subjects then performed a submaximal and maximal cycling protocol on a Velotron cycle ergometer (Racer Mate, Seattle, Washington, USA). The submaximal protocol consisted of 4 four-minute stages at 40, 80, 120, 160 watts (W) [24,30,31]. Following a 10-min recovery, subjects then performed a maximal cycle ergometer test that began at a work rate of 60 W for two minutes and increased 25 W every minute until the participant reached volitional fatigue. Within this study, these submaximal and maximal tests were used to quantify subject fitness (VO_2_ peak) and prescribe the cycling workloads for the phase one protocol [30,32,33]. Oxygen consumption was measured during both cycling tests via Parvo-Medics metabolic cart (Parvo-Medics, Sandy, Utah, USA). These two tests were used to quantify subject fitness (VO_2_ peak) and prescribe the cycling workloads for the phase one protocol.

### 2.4. Phase 1: Visits Two & Three

Visits two and three were identical with the exception that one occurred in normoxic conditions and the other occurred with 15% inspired O^2^ via hypoxia chamber (Altitude Control Technologies, Lafayette, CO, USA) simulating an altitude of 2740 m. Upon arrival into the laboratory, participants were seated in the hypoxia chamber, which was either turned on or off depending on the condition, and rested quietly for 30 min. During this time near infrared spectroscopy (NIRS) electrodes were secured to the skin over the vastus lateralis (approximately 15 cm above the proximal border of the patella and five centimeters lateral to the midline of the thigh) (Artinis Medical Systems, Oxymon MkIII, The Netherlands). Following the 30-min acclimation period heart rate, blood pressure, SaO2 and lactate were measured. Additionally, NIRS was used to obtain a measurement of muscle oxygenated hemoglobin, total hemoglobin as well as tissue saturation index (TSI) which is the ratio of oxygenated hemoglobin to total hemoglobin.

The experimental protocol consisted of two 16-min bouts of either single or double-leg cycling presented in random order. The 16-min bouts were composed of four 4-min stages. The workloads for the double-leg cycling were set to achieve 50%, 60%, 70% and 80% of the subject’s VO_2_ peak [24,30,31]. To maintain limb specific work rates, the workloads during single leg cycling were half that of double-leg. Subjects were instructed to maintain a pedaling rate of 80 rpm based on visual feedback from the Velotron software. There was a 30-s break between each stage to allow for femoral blood flow recording and a 15-min break between the single and double-leg protocols. During each stage, changes in oxyhemoglobin, total hemoglobin and TSI were recorded continuously. Heart rate, blood pressure, SaO2 and lactate were recorded during the last minute of each stage. Finally, femoral artery blood velocity and vessel diameter were measured at the end of each stage using a Logiq 7 Doppler/ultrasound machine with an M12 linear transducer (General Electric Medical Sytems, Milwaukee, WI, USA). Specifically, following the completion of each four-minute stage the participants were instructed to immediately extend their active leg and rest it on a box next to the ergometer. While participants remained seated, the ultrasound image of the femoral artery was obtained and blood flow was measured within 4–5 s of pedaling cessation. Marking the location for probe placement on the skin following baseline measurements aided in the quick transition from exercise cessation to probe placement. Once the vessel image was obtained, blood flow was measured for 10 s and then the participant began the next stage of cycling. Femoral blood flow was calculated in milliliters per minute based on blood velocity and arterial diameter utilizing the following equation: Blood flow = V_mean_ π (vessel diameter/2)^2^ × 60.

During the single-leg cycling a modified wooden box was placed directly next to the unoccupied crank arm in order to allow the participant to rest their inactive leg. A 10-kg counterweight was attached to a spindle on the crank arm opposite the active cycling leg which minimized the biomechanical differences between double-leg and single-leg cycling. Specifically, the counterweight assisted the active limb on the pedal upstroke, negating the need to recruit hip flexor muscles, while maintaining a smooth cycling motion similar to double-leg cycling [24,34]. Finally, only the right leg was used for single-leg cycling primarily due to the small space inside our hypoxia chamber and placement of ultrasound machine in relation to cycle ergometer which allowed easy access to the right femoral artery for blood flow measurements. For control purposes, the same researcher performed all of the femoral artery blood flow measurements.

### 2.5. Phase 2: Visits 2 and 3

Phase 2 focused on shorter bouts of maximal cycling exercise. These two visits were identical to each other with the exception that one was performed in normoxic conditions while the other was performed in hypoxic conditions. Upon arrival into the laboratory, participants were instructed to remain seated in the hypoxia chamber (which was turned on or off depending on condition) and rested quietly for 30 min to allow for acclimation. During this time, near infrared spectroscopy (NIRS) electrodes were secured to the skin over the vastus lateralis. Following the initial warm-up, they performed either a double-leg or single-leg 30 s maximal Wingate test which were assigned to the subjects in a counter balanced order. A 15-min recovery period separated the double-leg and single-leg Wingate tests. The resistance on the flywheel was set at 9% body weight for the double-leg trial and 5.4% body weight for the single-leg trial for both sexes (note: as single-leg Wingate has never been reported in adults, the 5.4% resistance was based on pilot studies within our lab in which 60% of the double-leg resistance [0.090 × 0.60 = 0.054] produced max power) [2,35,36,37,38,39,40]. The counterweight was utilized for the single-leg trial as described above. Throughout each 30 s Wingate protocol, tissue oxygenation, oxygenated hemoglobin, total hemoglobin, and TSI were recorded. Full recovery of the participant was determined by feedback from subjects and ensuring lactate, heart rate, and tissue oxygenation were back to baseline levels.

### 2.6. Data Analysis

The main goal of this investigation was to determine if the increased blood flow during single-leg cycling in hypoxia (SLH) can improve tissue oxygenation compared to double-leg cycling in hypoxia (DLH) such that it would be similar what is observed during double-leg cycling in normoxic conditions (DLN). Thus, the single-leg trial in normoxia, although performed to maintain consistency between the hypoxia and normoxia trials, was deemed not relevant and to maintain focus of the paper those data are not reported. The dependent variables that were assessed during the submaximal cycling protocols were heart rate, SaO2, lactate, mean arterial pressure (MAP), femoral artery blood flow, changes in oxyhemoglobin, total hemoglobin, and TSI. A paired samples *t*-test was used to compare pre-exercise heart rate, SaO2, lactate and MAP between normoxia and hypoxia conditions. A 3 × 4 repeated measures ANOVA was used to assess the main effects of condition (single-leg hypoxia, double-leg normoxia and double-leg hypoxia) and work rate (50%, 60%, 70%, and 80% of VO_2_ peak) as well as their interaction on all dependent variables. If the ANOVA was deemed significant, then a Sidak post hoc test was used to accommodate for alpha inflation. Assumptions of normality and sphericity were assessed and found no violations of these two assumptions. During the Wingate test oxygenated hemoglobin, total hemoglobin and TSI were recorded in 5 s intervals. A one-way repeated measures ANOVA were used to compare the following values between the three conditions: 1) average value across the entire 30 s Wingate, 2) average of first 5 s, 3) average of last 5 s, and 4) the change from the first five seconds to the final five seconds. If significance was found in the one-way repeated measures ANOVA, a post hoc test was used to determine where significance occurred. For all analysis alpha level of significance was set at *p* ≤ 0.05 and SPSS software was used for all statistical analyses (SPSS version 25, SPSS Inc., Armonk, NY, USA). All data were reported as mean ± standard deviation.

## 3. Results

### 3.1. Phase 1: Submaximal Results

Power

Double-leg power output during the 50%, 60%, 70%, and 80% aerobic stages were 113 ± 43, 145 ± 53, 178 ± 62, and 210 ± 71 W respectively. Single-leg power output during the 50%, 60%, 70%, and 80% aerobic stages was 56 ± 22, 73 ± 26, 89 ± 31, and 105 ± 35 W respectively.

Pre-exercise Values

The pre-exercise (resting) values for MAP, HR, RPE and lactate were not different between the hypoxic and normoxic condition (*p* ≥ 0.137 for all comparisons). However, SaO2 was reduced at rest in the hypoxic condition (95.7 ± 1.3%) prior to exercise compared to the normoxic condition (98.2 ± 0.63%) (*p* < 0.001).

Exercise
Femoral Blood Flow

**Figure 2:**
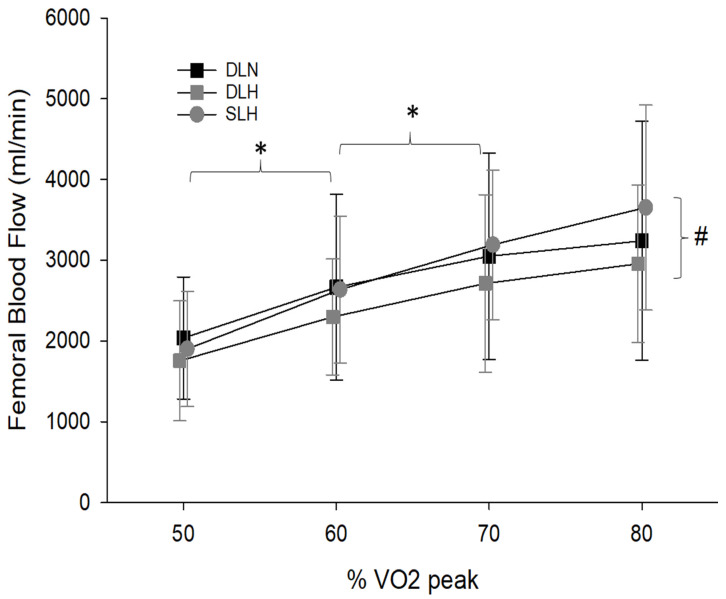
Changes in femoral blood flow between the three conditions and across the four different work rates. * Indicates the significant main effect of work rate on blood flow. Blood flow was not significantly different between 70% and 80% VO_2_ peak (*p* = 0.10). # Indicates a significant main effect (*p* = 0.02) between the single and double-leg trials in hypoxia. DLN = Double-Leg Normoxia; DLH = Double-Leg Hypoxia; SLH = Sigle-Leg Hypoxia.

Due to difficulty obtaining quality ultrasound images for two subjects, data for femoral blood flow represents eight of the 10 subjects. The 3 × 4 repeated measures ANOVA indicated a significant main effect of condition (F (2, 14) = 4.26, *p* = 0.036) and work rate (F (3, 21) = 31.95, *p* < 0.001) but not their interaction (F (6, 42) = 1.16, *p* = 0.345). In general, blood flow increased with higher exercise intensity in all conditions, and the average blood flow rate was significantly higher during SLH than DLH (*p* = 0.018). There was no significant difference between the SLH and the DLN nor between DLN and DLH (*p* ≥ 0.36; Figure 2). Significant comparisons for phase one variables are illustrated in Table 1.

Tissue Oxygenation

**Figure 3:**
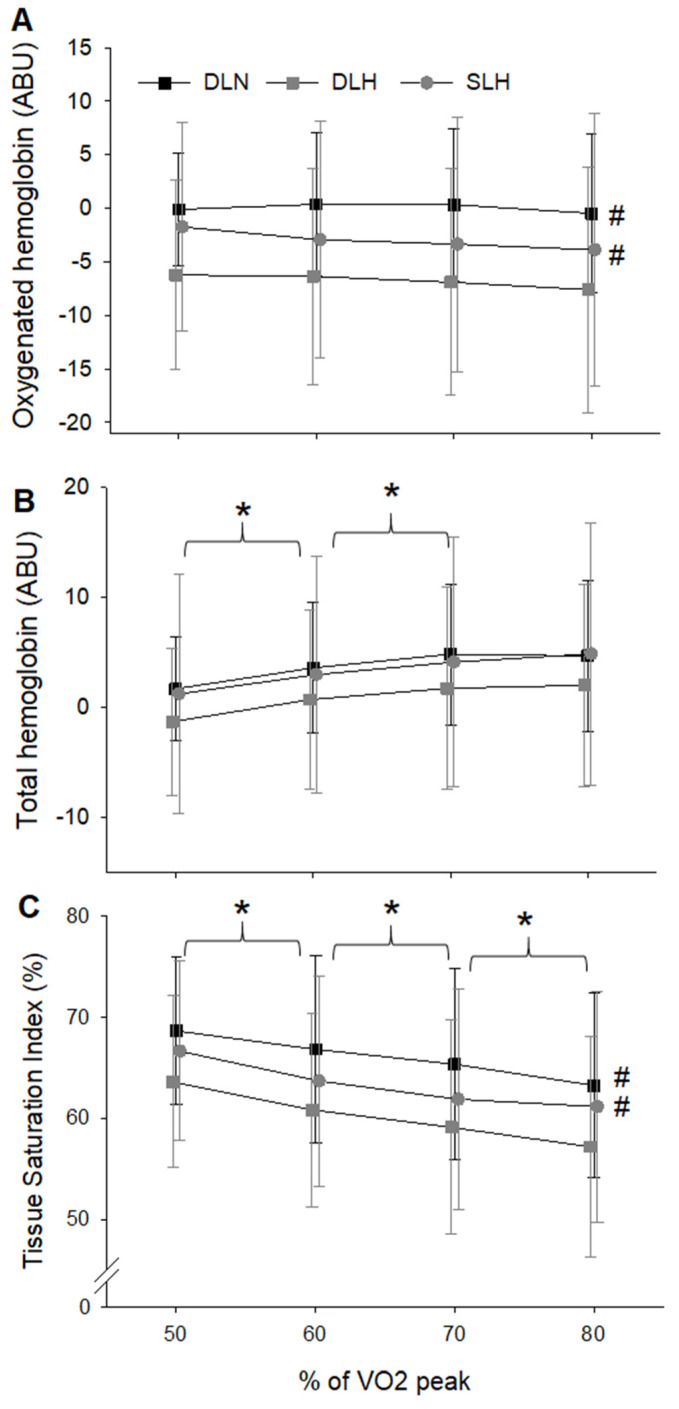
Changes in oxygenated hemoglobin (**A**), total hemoglobin (**B**) and TSI (**C**) across cycling condition and work rate. With regards to the main effect of condition, # indicates the significant reduction in both oxygenated hemoglobin and TSI during DLH compared to DLN (*p* ≤ 0.008) and SLH (*p* ≤ 0.01). Total hemoglobin was not significantly different across conditions (*p* = 0.458). With regards to the main effect of intensity, * indicates the significant change in total hemoglobin and tissue saturation with exercise intensity. TSI% decreased with each increase in work rate (*p* ≤ 0.011). DLN = Double-Leg Normoxia; DLH = Double-Leg Hypoxia; SLH = Sigle-Leg Hypoxia.

With regards to oxygenated hemoglobin there was a main effect of condition (F (2, 18) = 9.742, *p* = 0.001) but not work rate (F (3, 27) = 1.148, *p* = 0.348) nor their interaction (F (6, 54) = 1.625, *p* = 0.158). Oxygenated hemoglobin was significantly greater in the DLN (*p* = 0.002) and SLH (*p* = 0.01) compared to the DLH (Figure 3A). There was no difference in oxygenated hemoglobin between the DLN and SLH (*p* = 0.129). With regards to changes in total hemoglobin, there was a main effect of work rate (F (3, 27) = 16.914, *p* < 0.001) but not condition (F (2, 18) = 0.817, *p* = 0.458) nor their interaction (F (6, 54) = 0.232, *p* = 0.964). In general, total hemoglobin increased with each increase in work rate with the exception of 70–80% VO_2_ peak (Figure 3B). Finally, there was a main effect of condition (F (2, 18) = 7.96, *p* = 0.003) and work rate (F (3, 27) = 20.34, *p* = 0.001) but not their interaction (F (6, 54) = 0.6, *p* = 0.729) on TSI%. Specifically, TSI% was significantly reduced in the DLH cycling compared to DLN (*p* = 0.008) and SLH (*p* < 0.001) (Figure 3C). There was no difference between the DLN and SLH (*p* = 0.168). Furthermore, TSI% decreased with each increase in work rate (*p* ≤ 0.011) for all main effect comparisons across work rate.

Heart Rate and Mean Arterial Pressure

**Figure 4:**
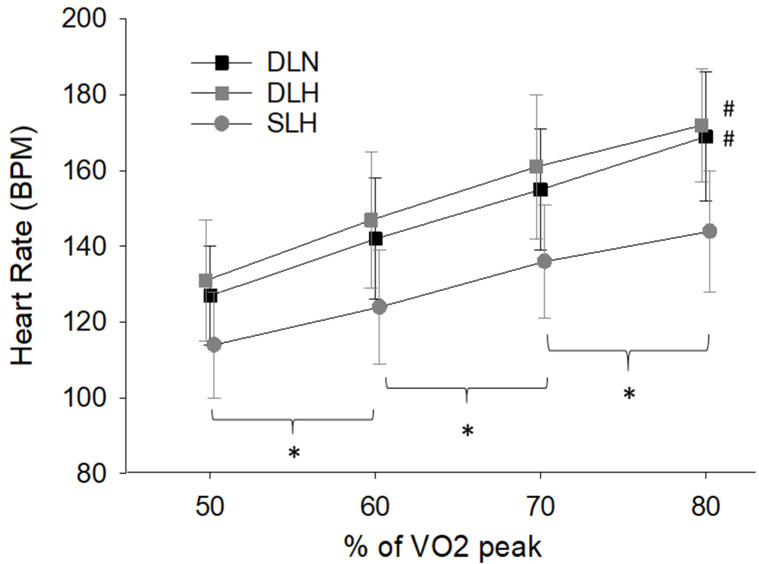
Changes in heart rate across the four different work rates. Heart rate was significantly lower during the single-leg cycling trial compared to the double-leg cycling trials (*p ≤* 0.004). There was no difference in heart between double-leg cycling in normoxia and hypoxia (*p* = 0.23). * Indicates the significant increase in HR with each stage of exercise intensity. # Indicates the main effect of both DLH and DLN were different than SLH. DLN = Double-Leg Normoxia; DLH = Double-Leg Hypoxia; SLH = Sigle-Leg Hypoxia.

There was a significant main effect of condition (F (2, 18) = 31.9, *p* < 0.001), work rate (F (3, 27) = 350.6, *p* < 0.001) and their interaction (F (6, 54) = 7.0, *p* < 0.001) on heart rate. In general, heart rate increased with each increase in work rate and heart rate was significantly lower during the single-leg cycling trial compared to both double-leg cycling trials (*p* ≤ 0.004). No difference in heart rate was found between the two double-leg cycling conditions (*p* = 0.23; Figure 4). There was also a significant main effect of work rate (F (3, 27) = 21.1, *p* < 0.01) but not condition (F (2, 18) = 3.3, *p* = 0.06) or their interaction (F (6, 54) = 0.77, *p* = 0.60) on MAP. Specifically, there was no difference in MAP when cycling at 50% or 60% of VO_2_ peak (97.0 ± 8.0 and 101.0 ± 10.0 mmHg, respectively, *p* = 0.13). However, MAP increased with every stage beyond 60% (70% = 104.9 ± 3.3 and 80% = 108.4 ± 3.7) (*p* ≤ 0.03 for all comparisons).

Arterial Oxygen Concentration

**Figure 5:**
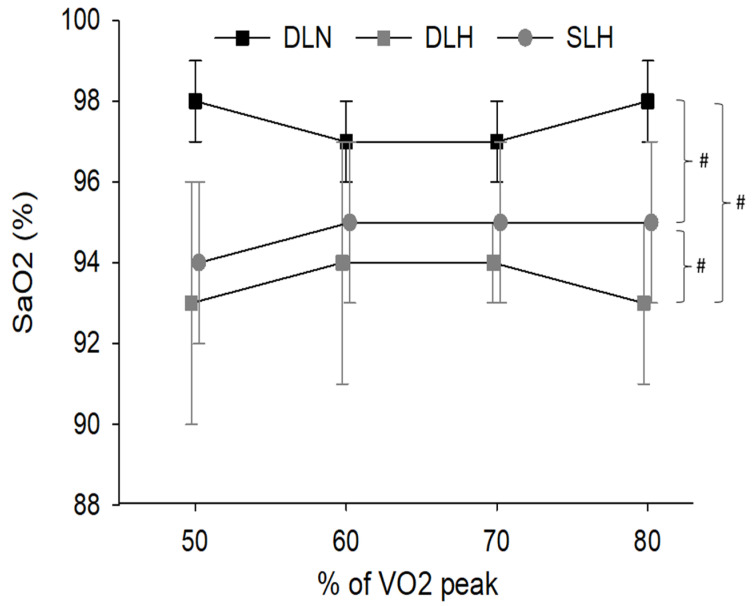
Changes in SaO2 concentrations across the four different work rates. # Indicates significantly different SaO2 concentrations for main effect of condition (*p* ≤ 0.05). DLN = Double-Leg Normoxia; DLH = Double-Leg Hypoxia; SLH = Sigle-Leg Hypoxia.

Cycling condition (F (2, 18) = 46.0, *p* < 0.01) but not work rate (F (3, 27) = 1.0, *p* = 0.41) nor their interaction (F (6, 54) = 0.91, *p* = 0.50) had an impact on SaO2. SaO2 was significantly lower in the SLH and DLH and trials compared to the DLN trial (*p* ≤ 0.01). However, SaO2 during the SLH trial was significantly higher than the DLH trial (*p* = 0.05; Figure 5).

Lactate

**Figure 6:**
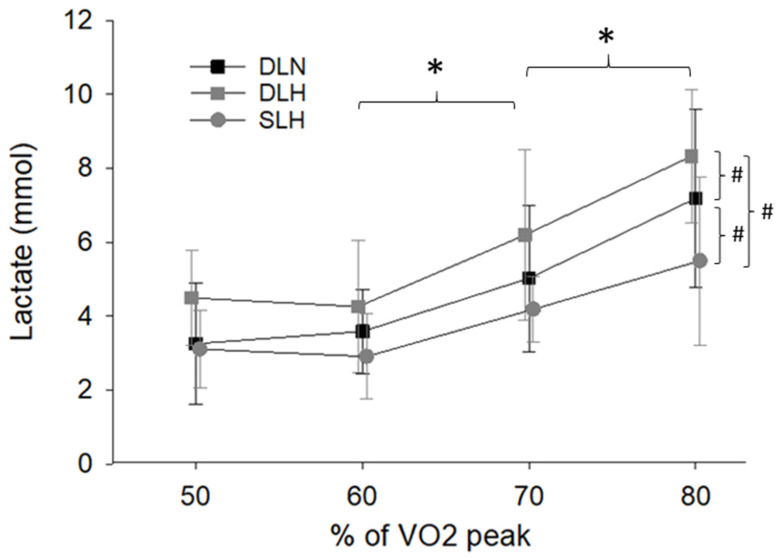
Blood lactate concentrations across the 4 submaximal cycling intensities and 3 cycling conditions lactate. * Indicates significant main effect of cycling intensity whereas # indicates a significant main effect of condition. Lactate increased from 60% to 70% VO_2_ peak and increased again from 70 to 80% VO_2_ peak. Lactate was also greater during double-leg cycling in hypoxia compared to double-leg cycling in normoxia. Lactate was lowest during the single-leg hypoxic condition. DLN = Double-Leg Normoxia; DLH = Double-Leg Hypoxia; SLH = Sigle-Leg Hypoxia.

Cycling condition (F (2, 18) = 19.93, *p* < 0.001) and work rate (F (3, 27) = 21.93, *p* < 0.001) had main effect on lactate but not their interaction (F (6, 54) = 1.05, *p* = 0.407) (Figure 6). Specifically, lactate was not significantly different between 50 and 60% VO_2_ peak (*p* = 0.903) but then increased from 60% to 70% VO_2_ peak (*p* = 0.003) and again from 70% to 80% VO_2_ peak (*p* < 0.001). Additionally, with regard to the main effect of condition, lactate during DLH was greater than DLN (*p* = 0.002) which was greater than SLH (*p* = 0.045).

### 3.2. Phase 2: Wingate Results

Power

The average power across the 30 s Wingate test was not significantly different between DLN (655 ± 140 W) and DLH (648 ± 146 W) but power during both double-leg conditions were significantly higher than SLH (357 ± 91 W) (*p* < 0.001).

Muscle Oxygenation Kinetics

**Figure 7:**
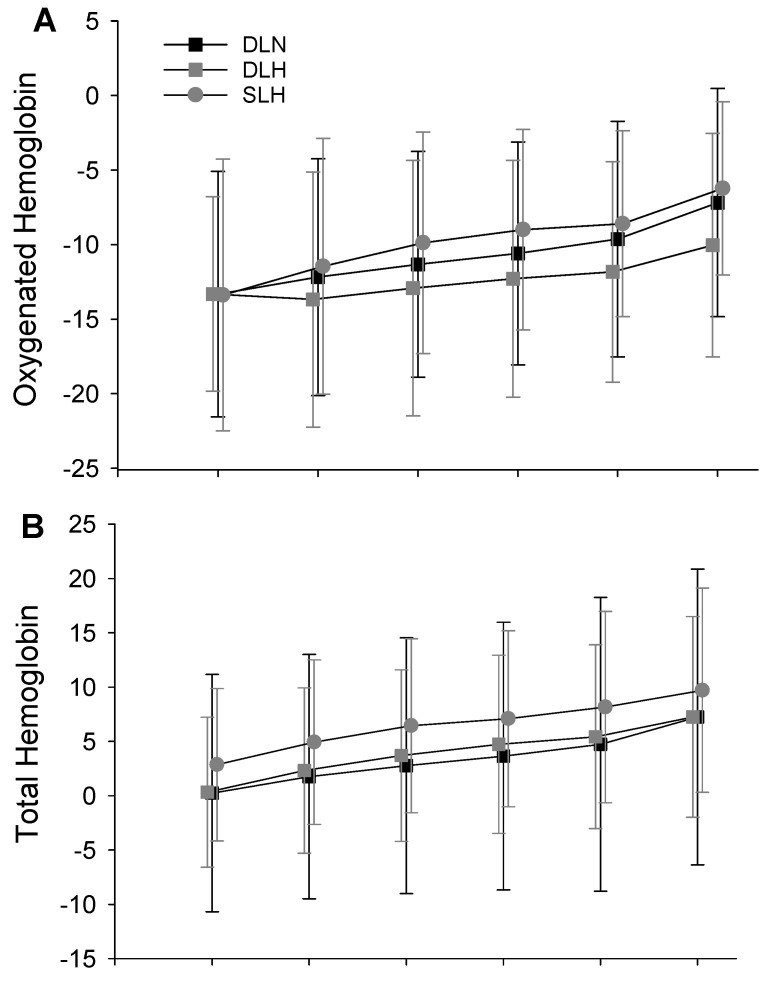

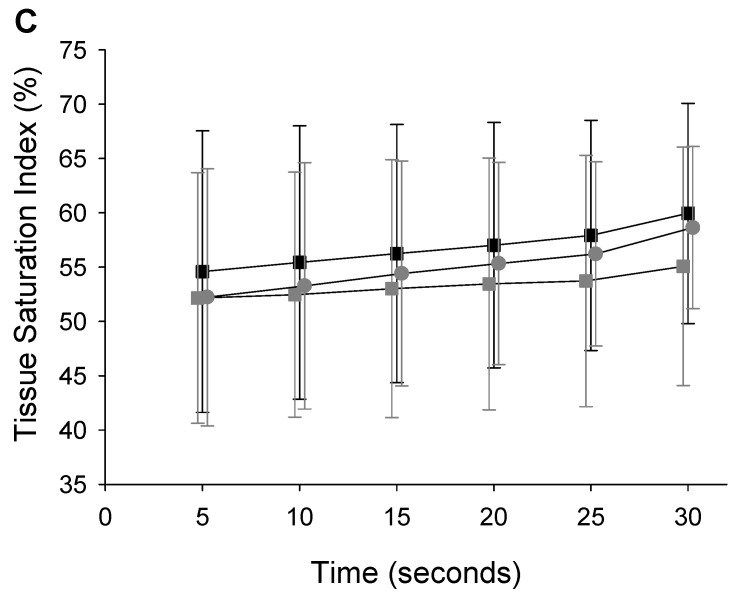
Five second averages of oxygenated hemoglobin (**A**), total hemoglobin (**B**) and TSI (**C**) during the 30 s maximal Wingate test. Comparing across the three conditions, there were no differences during the first 5 s, final 5 s, the delta from the first to last 5 s or the average across the entire 30 s test. DLN = Double-Leg Normoxia; DLH = Double-Leg Hypoxia; SLH = Sigle-Leg Hypoxia.

Unlike the submaximal protocol, during the 30-s maximal Wingate test there were no differences in oxygenated hemoglobin, total hemoglobin or TSI between the three conditions for the first 5 s, final 5 s, delta between the first five and last five seconds as well as the average values across the entire 30-s bout (Figure 7; for all comparison *p* ≥ 0.081).

## 4. Discussion

### 4.1. Main Findings

The main purpose of this investigation was to determine if the decrease tissue oxygen saturation during traditional double-leg cycling in hypoxic conditions could be offset by the increase in limb blood flow observed during single-leg cycling. Our data indicate that both oxygenated hemoglobin and total saturation index were reduced during double-leg submaximal cycling in hypoxic compared to normoxic conditions. The greater blood flow observed during single-leg cycling partially restored tissue oxygenation as there was no difference in oxygenated hemoglobin and TSI between the double-leg cycling in normoxia and single-leg cycling in hypoxia. Similar restoration of lactate and SaO2 also occurred during single-leg cycling in hypoxia compared to double-leg cycling. Although similar trends were observed during maximal cycling, the differences failed to achieve statistical significance. These finding partially confirms our hypothesis and provide support to the use of single-leg cycling as a potential means to improve muscle specific exercise intensity while living at altitude.

### 4.2. Physiological Changes during Sub-Maximal Cycling

During this investigation, blood flow during single-leg in hypoxia was significantly greater than blood flow during the double-leg cycling hypoxic condition. It is likely that at higher intensities (>80% VO_2_ peak) these differences in blood flow between the SL and DL conditions would increase as it appears that SL blood flow is diverging from DL blood flow as intensity increases. These findings support our hypothesis and confirm previous reports [24,31,34], in that reducing the active muscle mass allows for a greater percentage of cardiac output to be distributed to the active muscle.

Arterial oxygen concentration was found to be significantly higher in the double-leg normoxia trial compared to the single and double-leg hypoxia trials which is consistent with previous research and is a well-documented consequence of being in environments with reduced partial pressure of oxygen [41,42,43,44,45,46,47]. However, SaO2 during single-leg cycling in hypoxia was significantly higher than the double-leg cycling in hypoxia. This difference is likely due to a lower cardiac output during single-leg cycling resulting in a greater transit time for red blood cells to pick up oxygen in the pulmonary circulation and obtain more complete oxygen saturation [48,49,50]. 

With regard to the two hypoxic conditions these data support our hypothesis that greater femoral blood flow and SaO2 during single-leg cycling would result in greater tissue oxygenation and TSI in the active muscle (vastus lateralis). Furthermore, there was no difference in either TSI or oxygenated hemoglobin between SLH and DLN. Thus, these data support the notion that single-leg cycling could be used to help minimize the reduction in tissue oxygenation and ultimately limb-specific muscular work during cycle training at altitude. However, during this investigation the work performed by the active leg during single-leg cycling was equal to the work that leg performed during double-leg cycling. Specifically, external power produced during single-leg cycling was prescribed to be half of the power produced during double-leg cycling. Thus, the extra sustainable limb specific work that could be achieved during single-leg cycling in hypoxia, compared to double-leg cycling, needs to be investigated.

As expected, HR increased with work rate and was reduced during the single-leg cycling protocol. We expected heart rate to be lower during the single-leg trial in hypoxia due to the fact that external power produced during the double-leg cycling was twice that produced during single-leg cycling. This approach was different than our previous investigations during which total power output was similar between double and single-leg cycling, resulting in doubling of limb specific work rate during the single-leg cycling [24,30,31]. These previous reports indicated near similar HR and MAP between single and double-leg cycling when external power output is similar, suggesting that single-leg cycling, rather than traditional double-leg cycling, can also be utilized to maximize peripheral metabolic stress while maintaining near similar central cardiovascular load. 

The changes in lactate during the cycling protocols was as expected. In general, lactate increased as cycling intensity increased. In addition, lactate was highest while double-leg cycling in hypoxia and lowest during single-leg cycling. This agrees with previous reports indicating lactate production is greater during exercise at altitude compared to sea level [51,52,53,54,55]. In addition, during single-leg cycling, although the limb specific workload was similar to that of double-leg cycling, the overall workload was reduced by 50% and the greater blood flow and tissue perfusion to the active limb likely contribute to the reduced blood lactate concentration during the single-leg cycling condition.

### 4.3. Physiological Changes during Maximal Cycling

Although tissue perfusion and oxygenation varied during the submaximal cycling conditions, our data indicate that these differences did not transfer over to the 30-s maximal Wingate tests. Oxygenated hemoglobin, total hemoglobin and TSI were similar across all three cycling conditions during the Wingate test. This is not surprising due to the short duration of this test and greater reliance on non-aerobic energy systems including the ATP-PC system and anaerobic glycolysis. These results may have been different with a longer maximal test (i.e., 60 s) or during repeated maximal efforts interspaced with recovery periods (i.e., 30 s/1 min off).

### 4.4. Application and Limitations

These findings suggest that single-leg cycling may be a viable training modality that would at least partially offset the detrimental effects of the hypoxic environment during standard double-leg submaximal cycle training at altitude. Although not fully tested in this investigation, the increased blood flow and tissue oxygenation should enable individuals to train at sea level or greater intensity (i.e., limb specific power) while living at or visiting locations at altitude. A subsequent investigation should determine if the drop in limb specific sustainable cycling power due to altitude can be corrected with single-leg cycling.

There are limitations to the present study that should be noted. Having a larger and more homogeneous group in terms of sex and similar training level may reduce the variance observed across the variables collected in this investigation. The subjects in the present study were recreationally trained individuals, thus the current results cannot be extrapolated to highly trained athletes. However, it is likely that the benefits offered by single-leg cycling at altitude maybe more pronounced in highly trained athletes as they are more likely to exhibit signs of exercise induced hypoxemia during intense training [48]. It is also likely that we may have seen more pronounced benefits of single-leg cycling if the hypoxic conditions had a lower percent oxygen which would have simulated higher altitudes. However, we chose moderate altitude as the results would be more applicable to a greater number of recreational and elite athletes who live and train at moderate altitudes.

## 5. Conclusions

In conclusion, the results from this investigation suggest that elevated hemoglobin saturation and femoral blood flow during the single-leg condition in hypoxia are similar to that observed during double-leg cycling in normoxia. Additionally, based on the results of this investigation, single-leg cycling would prove to be a viable training modality that would offset the main disadvantage of living at altitude by enabling an individual to exercise at the similar submaximal levels of intensity achieved at sea level. 

## Figures and Tables

**Table 1:** Mean difference, 90% CI and effect size for significant comparisons.

	Mean Difference	95% CI	Cohen’s d		Mean Difference	95% CI	Cohen’s d
**FBF**				**SaO2**			
50%–60%	−637.2	−972.6; −301.8	0.714	DLH-DLN	−4.377	−5.57; −3.18	3.72
60%–70%	−451.9	−806.4; −97.4	0.418	SLH-DLH	1.535	0.026; 3.096	0.9
SLH-DLH	417.1	81.1; 753.2	0.427	SLH-DLN	−2.842	−4.117; −1.566	2.07
**MAP**				**Oxy**			
60%–70%	−4.117	−7.492; −0.741	0.399	DLN-DLH	6.809	3.177; 10.44	0.798
70%–80%	−3.467	−6.628; −0.306	0.308	SLH-DLH	−3.799	−6.422; 1.176	0.354
**HR**				**Total**			
50%–60%	−13.514	17.129; −9.899	0.955	50%–60%	−1.892	−2.568; −1.216	0.276
60%–70%	−13.117	−16.091; −10.142	0.859	60%–70%	−1.136	−2.093; −0.178	0.15
70%–80%	−11.094	−13.249; −8.940	0.723				
SLH-DLH	−23.21	−32.551; −13.870	1.475	**TSI**			
SLH-DLN	−18.737	−8.989; −28.486	1.25	DHL-DLN	−5.838	−9.758; −1.917	0.632
				DHL-SLH	−3.156	−4.231; −2.080	0.316
**Lactate**				50%–60%	2.517	0.717; 4.316	0.295
60%–70%	−1.553	−2.426; −0.681	1.2	60%–70%	1.668	0.881; 2.456	0.172
70%–80%	−1.867	−2.677; −1.057	1.104	70%–80%	1.581	0.895; 2.266	0.158
DLH-DLN	1.055	−1.615; −0.495	0.839				
DLN-SLH	−0.84	−1.655; −0.025	0.719				

## Data Availability

Data are contained within the article.
